# Self-activated surface dynamics in gold catalysts under reaction environments

**DOI:** 10.1038/s41467-018-04412-4

**Published:** 2018-05-25

**Authors:** Naoto Kamiuchi, Keju Sun, Ryotaro Aso, Masakazu Tane, Takehiro Tamaoka, Hideto Yoshida, Seiji Takeda

**Affiliations:** 1 The Institute of Scientific and Industrial Research, Osaka University, 8-1 Mihogaoka, Ibaraki, Osaka, 567-0047 Japan; 2 Research Institute for Ubiquitous Energy Devices, National Institute of Advanced Industrial Science and Technology (AIST), 1-8-31, Midorigaoka, Ikeda, Osaka, 563-8577 Japan; 3 Key Laboratory of Applied Chemistry, College of Environmental and Chemical Engineering, Yanshan University, 438 Hebei Avenue, Qinhuangdao, 066004 Hebei China

## Abstract

Nanoporous gold (NPG) with sponge-like structures has been studied by atomic-scale and microsecond-resolution environmental transmission electron microscopy (ETEM) combined with ab initio energy calculations. Peculiar surface dynamics were found in the reaction environment for the oxidation of CO at room temperature, involving residual silver in the NPG leaves as well as gold and oxygen atoms, especially on {110} facets. The NPG is thus classified as a novel self-activating catalyst. The essential structure unit for catalytic activity was identified as Au–AgO surface clusters, implying that the NPG is regarded as a nano-structured silver oxide catalyst supported on the matrix of NPG, or an inverse catalyst of a supported gold nanoparticulate (AuNP) catalyst. Hence, the catalytically active structure in the gold catalysts (supported AuNP and NPG catalysts) can now be experimentally unified in low-temperature CO oxidation, a step forward towards elucidating the fascinating catalysis mechanism of gold.

## Abstract (web summary)

Nanoporous gold (NPG) has gained significant attention, but its catalytically active structure has not yet been clarified. Here, the authors identify the catalytically active and dynamic structure in NPG by combining atomic-scale and microsecond-resolution environmental transmission electron microscopy with ab initio calculations.

## Introduction

Porous materials such as zeolites and porous silica are widely applied to catalysts and adsorbents because of their high surface area. In exploring higher surface areas with sufficient thermal durability, various porous materials have been exploited from metals to oxides. It is reported that nanoporous gold (NPG) with sponge-like structures^1–4^ acts as a catalyst to promote chemical reactions. Since gold is chemically inactive, the catalytic activity of NPG has gained significant attention in addition to conventional gold catalysts—that is, supported gold nanoparticulate (AuNP) catalysts^5–10^. However, the catalytically active structure has not yet been clarified in NPG. Several hypotheses have been proposed for the active structure in NPG. It was claimed that the specific structures that compose of atoms with low coordination number, for instance, those at steps, kinks, surface defects, twin boundaries, and dislocations^11–13^, play an important role for catalyzing chemical reactions. Other studies suggested that impurities in the system are responsible for catalytic performance^14–16^. NPG is generally prepared by a dealloying method of bimetallic alloys such as Au–Ag^17^ and therefore a small amount of impurity inevitably remains in final NPG catalyst. Deronzier et al.^18^ already attempted to fabricate a pure NPG by dissolving a Au_0.5_Zr_0.5_ alloy in hydrofluoric acid. This NPG, having significantly less residual Zr (0.03%), exhibited poor activity toward CO oxidation even at an elevated temperature of 140 °C, and therefore, the authors concluded that the residual impurity is indispensable for catalyzing CO oxidation reactions^18^. Haruta^19^ has suggested that NPG is an inverse catalyst of AuNP catalysts. Recently, Zugic et al.^20^ suggested that the surface restructuring of NPG, caused by ozone pretreatment followed by the reduction process in CO and/or CH_3_OH, resulted in metal oxide particles that covered the NPG surface up to a surface coverage of ~11%. They concluded that the oxygen in the specific state can catalyze the selective oxidation of CH_3_OH. However, the essentially active structure of NPG in the reaction environment has remained unclear. Here, we identify the catalytically active and dynamic structure in NPG by atomic-scale and microsecond-resolution environmental transmission electron microscopy (ETEM) combined with ab initio energy calculations. We show that for low-temperature CO oxidation, the active structure is self-organized on the surface of NPG in reaction environments. The result unifies experimentally the catalytically active structure of extended gold catalysts such as NPG and AuNP catalysts into heterogeneous nanostructures of gold and metal oxides.

## Results

### Surface dynamics in various environments

**Fig. 1:**
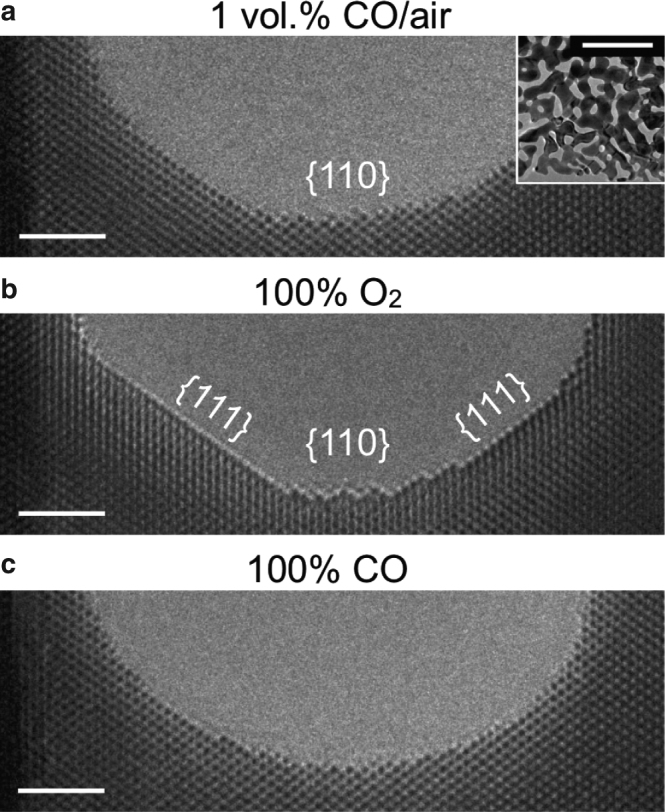
Drastic changes in surface morphology around a pore in NPG. **a** 1 vol.% CO/air (100 Pa), **b** 100% O_2_ (100 Pa), and **c** 100% CO (100 Pa). The inset in **a** reveals the typical porous structure of the NPG at low magnification. Scale bars: 2 nm, 200 nm (inset)

In this study, NPG was prepared from a commercially available Au–Ag alloy leaf by the same process as in previous works of catalyst preparation^3,11,21^ (Supplementary Note 1). A representative TEM image of a porous structure is shown in the inset of Fig. 1a. The amount of residual Ag was estimated below 1.0 at.% by quantitative analyses of TEM-energy dispersive X-ray spectroscopy (EDX) and X-ray photoelectron spectroscopy (XPS) measurements (Supplementary Tables 1 and 2). No other impurity metal elements were detected at all within the limits of accuracy of apparatuses. The as-prepared NPG has pores of size ranging from 10–100 nm (Supplementary Fig. 1) (typically ~15 nm). The pores exhibit round surfaces partially covered with {111}, {100}, and {110} facets, as is shown in Supplementary Fig. 2. To confirm that ETEM observation in the NPG specimen is not induced by residual impurities other than Ag in the commercially available Au–Ag alloy leaf, we also prepared a NPG specimen from a well-defined starting alloy of Au–Ag by melting pure Au and pure Ag and performed ETEM observation, as is summarized in Supplementary Note 1. The direct electron detection system by TEM^22^ allows atomic-scale and microsecond-resolution ETEM to clarify atomic and molecular dynamics in one cycle of a chemical reaction. Indeed, ETEM in this study was performed using both a high-speed TEM camera (K2 IS Direct Detection Camera, Gatan, Inc., USA)^22^ and an atomic resolution ETEM apparatus (Titan ETEM G2 equipped with a specially designed environmental cell, a spherical aberration corrector of the objective lens^23^, and a monochromator^24^) (Supplementary Note 2). Therefore, the catalytically active and dynamic structure in one catalytic cycle can be revealed for the first time by atomic-scale and microsecond-resolution ETEM.

Atomic-scale and microsecond-resolution ETEM images of NPG in various environments are summarized in Fig. 1. While the surface appeared to be smooth and round in pure CO (100 Pa) (Fig. 1c), the {111} facets are dominant in pure O_2_ (100 Pa) (Fig. 1b) with associated peculiar {100} and {110} nanofacets. The drastic changes in surface morphology indicate that gas molecules of O_2_ strongly interact with the surface atoms. We succeeded in capturing the peculiar surface dynamics under a reaction environment for the oxidation of CO that includes both O_2_ and CO (1 vol.% CO/air (100 Pa)). The dynamics are self-activated on an extended {110} nanofacet (Fig. 1a). It is noteworthy that the environment-dependent change in surface morphology is reproduced across any surface type—either concave, convex or saddle-shaped areas of NPG (Supplementary Note 3, Supplementary Figs. 7 and 8). The environment-dependent change in surface morphology is also reproduced at a higher gas pressure of 1000 Pa (Supplementary Note 1, Supplementary Fig. 3). Furthermore, the change is reproduced in a NPG specimen from a well-defined starting alloy of Au–Ag by melting pure Au and pure Ag (Supplementary Note 1, Supplementary Fig. 6). Therefore, the change could be attributed to the difference of environments, although in ETEM observation, the surfaces are irradiated by high-energy electrons.

### Identification of self-activating nanofacets

**Fig. 2:**
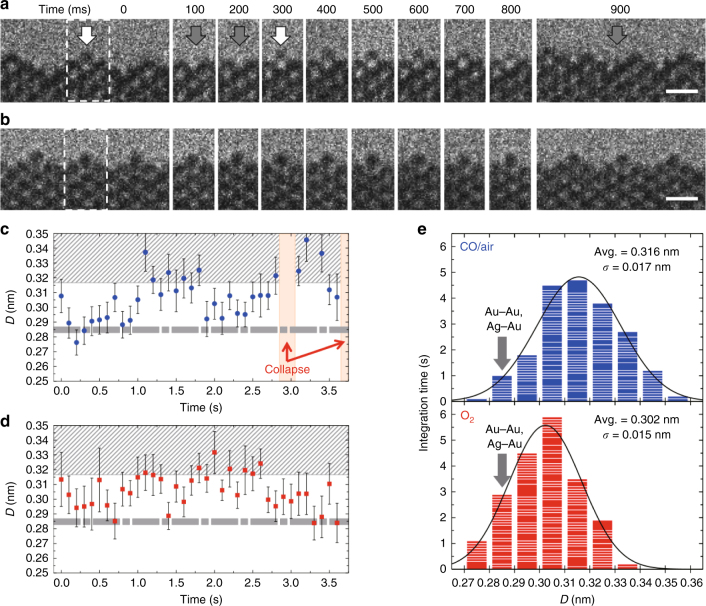
Dynamic nature of a self-activating nanofacet on {110}. A nanofacet being **a** dynamic in CO/air (1 vol.% CO/air, 100 Pa) and **b** stable in O_2_ (100% O_2_, 100 Pa). Images are taken from in situ environmental transmission electron microscopy (ETEM) movies (frame rate: 40 fps) (Supplementary Movies 1 and 2). In **a**, white arrows indicate the top atomic column of the nanofacet, and gray arrows at 100, 200, and 900 ms indicate the absence of atoms at the top atomic column, or displaced to an adjacent site (out of view) (Supplementary Movie 1). Note that the image contrast of the top atomic column varied with time (from 400 to 800 ms) (see also Supplementary Note 4). Time-dependent distances between the top atomic column and the atomic column immediately below, *D* in **c** CO/air and **d** O_2_. *D* was evaluated by Gauss fitting for intensity profiles of ETEM images (Supplementary Note 2). The fitting errors are shown as error bars. In **c**, **d**, dotted bars represent the range of *D* in pure gold and Au–Ag nanofacet models without oxygen, while shaded areas represent those Au–Ag nanofacet models with oxygen (or Au–AgO models). **e** Distribution of *D* for time intervals of 100 ms in CO/air and O_2_. Arrows in **e** indicate the corresponding distance between Au and Au atomic columns in pure crystalline gold surfaces (*D*_Au_ = 0.288 nm) and that between Au and Ag atomic columns in the nanofacet models (*D* = 0.2842–0.2849 nm) (Supplementary Note 5). In **a**, **b**, ETEM images at 0 and 900 ms are shown in full size, and those from 100–800 ms are shown partially in the area between the dotted lines at 0 ms. The image contrast and frame rate in Supplementary Movies 1 and 2 were adjusted with 4 × 4 × 4 binning. Scale bars: 0.5 nm

Figure 2a clarifies the dynamic nature of the nanofacet on {110} in the presence of the reaction environment (Supplementary Movie 1). The nanofacets sometimes collapse before reorganizing at a neighboring site and occasionally recovering at the initial site. For instance in Fig. 2a, the top atomic column at *t* = 0 s, indicated by the white arrow, disappeared at *t* = 100 ms, re-appeared from 300 to 400 ms and then disappeared gradually from 500 to 900 ms. The image contrast of the top atomic column gradually changes as a function of time in the reaction environment (Supplementary Note 4). The peculiar instability can only be accounted for by the dynamic interaction of surface atoms and gas molecules undergoing the catalyzed chemical reaction. As is confirmed in Fig. 2b and Supplementary Movie 2, the nanofacet is significantly stabilized in pure O_2_ without any drastic structural reorganization even over prolonged times. It is emphasized that the image contrast of the top atomic column remained largely unchanged. Since NPG includes a small amount of residual silver, the stable nanofacet originates from the oxidation of surface silver at the atomic scale. To quantify dynamic ETEM observation data, the distances between the top atomic column and the atomic column immediately below, *D* was quantitatively measured (Supplementary Note 2) within the temporal resolution of 100 ms, as is summarized in Fig. 2c, d for the reaction environment and pure O_2_, respectively. The time-dependent distance, *D* exceeds very frequently the corresponding distance in crystalline pure gold surfaces, *D*_Au_ = 0.288 nm or 1/2[110]*a* (*a* being the lattice parameter of fcc Au). *D* varies abruptly but somewhat periodically up to 0.36 nm in the reaction environment, while in O_2_, *D* varies rather randomly above 0.27 to 0.34 nm. Figure 2e summarizes the distribution of *D* for time intervals of 100 ms in the reaction environment and pure O_2_. The distribution in the reaction environment diverges more than that in O_2_, as indicated by the larger standard deviation. The larger divergence in the reaction environment reflects the structural reorganization and the significant fluctuation in the image contrast at the top atomic column, as is revealed in Fig. 2a. Possible structural models for the nanofacet, including the residual impurity of silver and adsorbed oxygen atoms, were built and evaluated energetically by means of ab initio calculations (refer Supplementary Note 5, Supplementary Fig. 10). The distance, *D* was not varied largely, only by substituting Ag atoms for Au atoms in the nanofacet model of pure gold (*D* = 0.2842–0.2849 nm), for instance, for those at the top atomic column. However, *D* is increased significantly (*D* = 0.3162–0.3519 nm) by the addition of oxygen atoms at the adsorbed sites of the Ag and/or Au atoms on the surface of the nanofacet models. The calculation results support the assumption that the fluctuation of *D* is induced by the adsorption and desorption of oxygen on nanofacets. Furthermore, the surface reorganization was reproduced in other surfaces in the convex area of NPG in the reaction environment (Supplementary Note 3, Supplementary Fig. 8). Additionally, we confirmed, for a pure Au foil without any catalytic activity (refer Supplementary Note 6), that no peculiar surface restructuring that led to nanofacets occurred, when subjected to the same NPG catalyst reaction environment (1 vol.% CO/air (100 Pa)). Ordinary and stable {111} facets were only observed, although the surface gold atoms moved through their interaction with CO^25^, as is seen in Supplementary Fig. 11a, b.

The catalytic activity of NPG can now be best described in light of the present ETEM observations. Surface Ag atoms in a nanofacet are spontaneously oxidized, resulting in atomic Ag–O clusters of higher structural stability in 100% O_2_, as illustrated in Fig. 2b. It has been suggested by both experimental^14,16,26^ and theoretical studies^15^ that elemental Ag facilitates the adsorption and dissociation of gaseous O_2_ molecules, i.e., activating oxygen species. Therefore, O atoms in the atomic Ag–O clusters on nanofacets are significant for the catalytic activity of NPG. Conversely, CO molecules strongly interact with the surface atoms of NPG. In 100% CO, pores become rounder regardless of facet type (Fig. 1c). Surface atoms migrate much more frequently in 100% CO than those in the reaction environments. Furthermore, it has been reported that the surface Au atoms of an Au {111} model catalyst become mobile even at room temperature by forming Au–CO complexes in 100% CO gas at low pressure (10^−8^–10^−4^ Torr)^27^. Therefore, it is most likely that CO molecules, adsorbed on the surface of NPG encounter O atoms derived from nanofacet atomic Ag–O clusters. The breaking of Ag–O bonds at the reaction with CO molecules results in the surface atom dynamics, as is shown in Fig. 2. Hence, nanofacets of only a few atomic columns wide that extend along <110> are the catalytically active structure of NPG for CO oxidation. Notably, ETEM provides us with only a two-dimensional projection of the dynamic nanofacet in the reaction environment. Our ETEM results show that the image contrast of the top atomic column varied with time (Supplementary Note 4). This implies that a segment of an atomic column acts as the catalyst for the chemical reaction, and not the entire atomic column. In addition to the major Au–AgO nanofacets, the characteristic extended structures appeared, however, this was infrequent (refer Supplementary Note 7). Both the single- and double-atomic layer structure types could be identified as AgO (Supplementary Figs. 14 and 15). This is consistent with the result from a study of NPG pretreated by ozone and followed by reduction in CO and/or CH_3_OH^20^. The catalytically active Au–AgO nanofacet in NPG, which is a dynamic nanostructure in atomic scale under the reaction environment, provides insight into the essentially active structure of AuNP catalysts. It is already known that a few atomic columns at the perimeter interface between a Au nanoparticle and a metal (M, typically titanium or cerium) oxide support are not structurally rigid in the reaction environment created by ETEM^10^. Therefore, it is most likely that dynamic Au-MO_*x*_ nanofacet-like structures are formed at the perimeter interface. In this respect, the Au–AgO nanofacet in the NPG may be regarded as an inverse catalyst of the AuNP catalyst^19^.

**Fig. 3:**
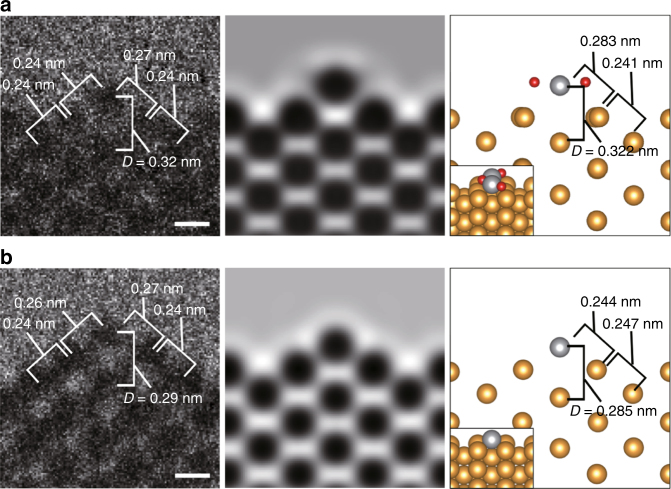
Au–AgO nanofacets as the essential atomic structure in NPG. **a** A typical nanofacet stabilized by silver atoms located at the top atomic column with oxygen atoms in 100% O_2_ (100 Pa) and **b** the nanofacet at the moment of instant desorption of oxygen atoms for the oxidation of CO molecules in 1 vol.% CO/air (100 Pa). From left to right in **a**, **b** are ETEM images, simulated images, and the models after ab initio calculations, respectively. Gold, silver, and red color balls represent Au, Ag, and O atoms, respectively. Quantitatively measured *D* in ETEM images agree well with those in the models, as seen in **a**, **b**. Distances between the inter-atomic columns near the top atomic columns are also shown. Scale bars: 0.2 nm

As is mentioned above, the image contrast of the nanofacets remains largely unchanged in O_2_ (Fig. 2b). This implies that heavy metals (Au and Ag), which mainly contribute to image contrast, remain at the same sites. Conversely, the nanofacet dynamics, involving the surface metal atoms in the presence of the reaction environments are reproduced when the electron current density is significantly lowered to 0.4 A cm^−2^ (from typically 4 A cm^−2^) (Supplementary Note 8, Supplementary Fig. 16). The nanofacet dynamics were never induced on pure Au surfaces (Supplementary Fig. 11). Therefore, the dynamics are regarded as an intrinsic phenomenon in reaction environments for CO oxidation in NPG catalysts. Comparing the measured *D* value (0.32 nm) for a typically stable nanofacet in O_2_ (Fig. 3a) with the calculated *D* in the models (Supplementary Note 5, Supplementary Fig. 10), the best fit is achieved for the model with the top atomic column fully occupied by Ag atoms that are associated with adsorbed O atoms (Au–AgO nanofacet model, right panel of Fig. 3a). The simulated image of the model (middle panel of Fig. 3a) agrees well with the observed image (left panel of Fig. 3a). A typical ETEM image representative of a smaller *D* value (0.29 nm) in the reaction environment (Fig. 3b) can be accounted for by removing O atoms from the Au–AgO nanofacet model for the oxidation of CO molecules (right panel of Fig. 3b). Image matching by ETEM observations combined with ab initio calculations in Fig. 3 further supports the assumption that in the presence of residual Ag atoms the adsorption and desorption of oxygen occurs at the top atomic column in the self-activating nanofacets for CO oxidation at room temperature. The possibility of other nanofacet models with a complex distribution of surface Ag atoms (Supplementary Note 5, Supplementary Fig. 10) could not strictly be ruled out, because the measured value of *D* diverged over *D*_Au_ = 0.288 nm and below 0.33 nm, and since in situ ETEM can only provide a two-dimensional projection of NPG surface in gas, or a projection of the surface atomic columns along the observation direction.

As is shown (Fig. 2b, d, e), *D* in the stable nanofacets in a pure O_2_ atmosphere is also varied without the restructuring of the surface metal atoms. This variation is attributed to the spontaneous oxidation and the artificial reduction of atomic Ag–O clusters. The latter is induced by electron beam irradiation. Nanofacets fluctuate between a structure with oxygen (i.e., right panel of Fig. 3a) and the corresponding structure without oxygen (i.e., right panel of Fig. 3b) by the balance of oxidation and reduction. As a further technical note in ETEM, amorphous layers (e.g., Fig. 6b of ref. ^11^, Fig. 3a of ref. ^21^) are often observed on the surface of NPG. As discussed in the Supporting Information (Supplementary Note 9, Supplementary Figs. 17–19), the formation of amorphous layers is an artifact by electron beam irradiation.

### Stability of nanofacets under a reaction environment

The stability and reversibility of the surface structural changes are critical issues, because the properties directly link to the durability of catalytic performance. It has been reported that the catalytic activity of NPG gradually degrades as a function of time^21^. To confirm the stability of surface structure and morphology, representative pores were observed in a reaction environment (1 vol.% CO/air (100 Pa)) for prolonged times. After confirming that nanofacets are gradually self-activated on the pore surface in the presence of the reaction environment (Supplementary Note 10, yellow squares in Supplementary Fig. 20b, c), the nanofacets slowly reorganized into smooth {110} surfaces without nanofacets after 14 h (Supplementary Fig. 20d). Metal atoms on and near the surface are topologically accessible to any part of the entire NPG surface. Long-range diffusion of surface atoms over prolonged times results in changes to microscopic surface morphology toward the energetically favorable morphology. Hence, the low durability of the NPG catalyst for CO oxidation is caused by the irreversible structural and morphological changes of the surface. Conversely, Au atoms in AuNP catalysts are isolated in a Au nanoparticle, and therefore the degradation is minimal for CO oxidation at room temperature.

## Discussion

For low-temperature CO oxidation in NPG, the estimated turnover frequency (TOF) ranges extensively from 1 × 10^−3^ to 1 × 10 s^−1^ (refer Supplementary Note 1). This range phenomenologically corresponds to the average time for catalysis in individual catalytic atomic sites from 1000 to 0.1 s. An elemental chemical reaction is much faster than the time scale of the ETEM observation. Nevertheless, during and/or after a single catalytic cycle of a heterogeneous catalyst such as the Au catalysts, it is likely that the catalytic structure reorganized at the atomic scale and an intermediate atomic structure is formed with a relaxation time that is commensurate with the time scale of the ETEM observation, for instance, the cluster of Au–AgO and that of Au–Ag without O. Therefore, it is possible to propose in this study that the atomic structures of intermediates are revealed in one cycle of a catalytic chemical reaction.

In conclusion, the NPG catalyst is classified as a novel self-activating catalyst. The drastic displacement of surface atomic columns is induced under reaction environments for the oxidation of CO (1 vol.% CO/air at 100 Pa at room temperature). By atomic-scale and microsecond-resolution ETEM combined with ab initio energy calculations, the peculiar surface dynamics were revealed involving residual silver as well as gold and oxygen atoms, especially on {110} facets. The essential structure unit for catalytic activity was identified as Au–AgO nanofacets. The nanostructure is regarded as a Ag oxide catalyst supported on the matrix of NPG, or an inverse catalyst of a AuNP catalyst. Therefore, the active structure of gold catalysts (AuNP and NPG catalysts) can now be discussed consistently in low-temperature CO oxidation and possibly other chemical reactions toward elucidating the fascinating catalysis mechanism of gold. The atomic structures of NPG and AuNP catalysts could be unified, although questions remain concerning the active oxygen species for chemical oxidation reaction such as CO oxidation and selective methanol oxidation. Finally, our finding indicates that it will be crucial to elucidate the self-activated and dynamic structure units for developing not only gold catalysts but industrial solid catalysts more efficiently in the immediate future.

## Methods

### Sample preparation

NPG was prepared from commercially available Au–Ag alloy leaves (Giusto Manetti Battiloro S.p.A, Italy) by a dealloying method. The nominal composition of the Au–Ag alloy leaves was Au/Ag = 40/60 wt.%, and the thickness was estimated to be about 140 nm. To prepare a NPG leaf, a Au–Ag alloy leaf was dealloyed for 3 h in 70% HNO_3_ aq., followed by rinsing sufficiently in distilled water. Well-defined NPG sample was also prepared by the same dealloying method of a Au–Ag starting alloy. For the Au–Ag starting alloy, pure Ag and Au grains were melted in a vacuum arc-melting furnace (ACM-01, Daia Vacuum Co., Japan). See Supplementary Information for experimental details.

### Characterization

The atomic composition of NPG sample was estimated by the quantitative analyses of EDX equipped with a TEM (JEM-ARM200F, JEOL Ltd., Japan) and XPS (PHI5000, ULVAC-PHI, Inc., Japan). The elemental analysis on the Au–Ag starting alloy was performed by XPS and inductively coupled plasma atomic emission spectroscopy (ICPE-9820, Shimadzu Co., Japan). To identify the amorphous layers formed on NPG surface in 100% O_2_, scanning transmission electrom microscopy—electron energy loss spectroscopy was performed at 300 keV (Titan ETEM G2, FEI company, USA). See Supplementary Information for experimental details.

### ETEM observation and image simulation

A part of an as-prepared NPG leaf was placed onto a Cu grid with a holey carbon supporting film. NPG was observed using an ETEM apparatus (Titan ETEM G2, FEI company, USA) with a spherical aberration corrector (Cs-corrector) of the objective lens, a monochromator and a K2 IS Direct Detection Camera (Gatan, Inc., USA). To minimize electron beam damage to NPG, the accelerating voltage and electron current flux during ETEM observations were set at 80 kV and 4 A cm^−2^, respectively. The spherical aberration of the objective lens was corrected to below 1 μm using the Cs-corrector. ETEM images were acquired at a frame rate of 40 fps in counting (summit) mode using a K2 camera. Three gases, 1 vol.% CO/air (1 vol.% CO, 21 vol.% O_2_, 78 vol.% N_2_) (100 Pa), 100% O_2_ (100 Pa), and 100% CO (100 Pa) were introduced into the specially designed environmental cell of the ETEM apparatus at room temperature. In the ETEM observation of the NPG sample formed from well-defined Au–Ag alloy, the NPG specimens were observed with a Ceta2 camera at 300 kV and 4 A cm^−2^ at the frame rate of 2 fps.

TEM images of model structures, constructed by ab initio calculation were simulated using MacTempasX software (Total Resolution, USA).

### Density functional theory calculation

Several Au/AgO_*x*_ surface models were constructed for a nanofacet on {110} and extended AgO atomic layers on {111}. For the structure optimization and energy calculation for energetically favorable structures in Au/AgO_x_, Vienna Ab-Initio Simulation Package (VASP) was used for the density functional theory calculations. See Supplementary Information for details.

### Data availability

The data that support the findings of this study are available from the corresponding author upon reasonable request.

## Electronic supplementary material

Supplementary Information

Description of Additional Supplementary Files

Supplementary Movie1

Supplementary Movie2

## References

[1] Forty AJ (1979). Corrosion micro morphology of noble metal alloys and depletion gilding. Nature.

[2] Erlebacher J, Aziz MJ, Karma A, Dimitrov N, Sieradzki K (2001). Evolution of nano porosity in dealloying. Nature.

[3] Ding Y, Erlebacher J (2003). Nanoporous metals with controlled multimodal pore size distribution. J. Am. Chem. Soc..

[4] Zielasek V (2006). Gold catalysts: nanoporous gold foams. Angew. Chem. Int. Ed. Engl..

[5] Haruta M, Kobayashi T, Sano H, Yamada N (1987). Novel gold catalysts for the oxidation of carbon monoxide at a temperature far below 0 °C. Chem. Lett..

[6] Haruta M, Yamada N, Kobayashi T, Iijima S (1989). Gold catalysts prepared by coprecipitation for low-temperature oxidation of hydrogen and of carbon monoxide. J. Catal..

[7] Fu Q, Saltsburg H, Flytzani-Stephanopoulos M (2003). Active nonmetallic Au and Pt species on ceria-based water-gas shift catalysts. Science.

[8] Kuwauchi Y, Yoshida H, Akita T, Haruta M, Takeda S (2012). Intrinsic catalytic structure of gold nanoparticles supported on TiO_2_. Angew. Chem. Int. Ed. Engl..

[9] Yoshida H (2012). Visualizing gas molecules interacting with supported nanoparticulate catalysts at reaction conditions. Science.

[10] Kuwauchi Y (2013). Stepwise displacement of catalytically active gold nanoparticles on cerium oxide. Nano Lett..

[11] Fujita T (2012). Atomic origins of the high catalytic activity of nanoporous gold. Nat. Mater..

[12] Kameoka S, Tanabe T, Miyamoto K, Tsai AP (2016). Insights into the dominant factors of porous gold for CO oxidation. J. Chem. Phys..

[13] Liu P (2016). Visualizing under-coordinated surface atoms on 3D nanoporous gold catalysts. Adv. Mater..

[14] Wittstock A (2009). Nanoporous Au: an unsupported pure gold catalyst?. J. Phys. Chem. C.

[15] Moskaleva LV (2011). Silver residues as a possible key to a remarkable oxidative catalytic activity of nanoporous gold. Phys. Chem. Chem. Phys..

[16] Wang LC, Zhong Y, Widmann D, Weissmüller J, Behm RJ (2012). On the role of residual Ag in nanoporous Au catalysts for CO oxidation: a combined microreactor and TAP reactor study. ChemCatChem.

[17] Ding Y, Kim YJ, Erlebacher J (2004). Nanoporous gold leaf: “ancient technology”/advanced material. Adv. Mater..

[18] Deronzier T, Morfin F, Massin L, Lomello M, Rousset JL (2011). Pure nanoporous gold powder: synthesis and catalytic properties. Chem. Mater..

[19] Haruta M (2007). New generation of gold catalysts: nanoporous foams and tubes—is unsupported gold catalytically active. ChemPhysChem.

[20] Zugic B (2017). Dynamic restructuring drives catalytic activity on nanoporous gold–silver alloy catalysts. Nat. Mater..

[21] Fujita T (2014). Atomic observation of catalysis-induced nanopore coarsening of nanoporous gold. Nano Lett..

[22] Li X (2013). Electron counting and beam-induced motion correction enable near-atomic-resolution single-particle cryo-EM. Nat. Methods.

[23] Takeda S, Kuwauchi Y, Yohida H (2015). Environmental transmission electron microscopy for catalyst materials using a spherical aberration corrector. Ultramicroscopy.

[24] Tiemeijer PC, Bischoff M, Freit M, Kisielowski C (2012). Using a monochromator to improve the resolution in TEM to below 0.5 A. Part I: Creating highly coherent monochromated illumination. Ultramicroscopy.

[25] Min BK, Alemozafar AR, Biener MM, Biener J, Friend CM (2005). Reaction of Au(111) with sulfur and oxygen: scanning tunneling microscopic study. Top. Catal..

[26] Wang LC, Friend CM, Fushimi R, Madix RJ (2016). Active site densities, oxygen activation and adsorbed reactive oxygen in alcohol activation on npAu catalysts. Faraday Discuss..

[27] Wang J (2016). Formation, migration, and reactivity of Au-CO complexes on gold surfaces. J. Am. Chem. Soc..

