# Supplementary material for: Self-activated surface dynamics in gold catalysts under reaction environments
Source: Nat Commun. 2018 May 25;9:2060. doi: 10.1038/s41467-018-04412-4 (PMC5970267; doi:10.1038/s41467-018-04412-4)
Supplement: Supplementary file 1 — Supplementary Information [file 41467_2018_4412_MOESM1_ESM.pdf]

Supplementary Information

Self-Activated Surface Dynamics in Gold Catalysts under Reaction Environments

Kamiuchi *et al.*

Supplementary Note 1. Preparation, quantitative analyses, and catalytic performance of NPG.

NPG was prepared from commercially available Au-Ag alloy leaves (Giusto Manetti Battiloro *S.p.A*, Italy) by a dealloying method[1]. The nominal composition of the Au-Ag alloy leaves was Au/Ag = 40/60 wt.%, and the thickness was estimated to be about 140 nm. To prepare a NPG leaf, a Au-Ag alloy leaf was dealloyed for 3 h in 70% HNO_3 *aq.*, followed by rinsing sufficiently in distilled water. The atomic composition of both a Au-Ag alloy leaf and a NPG leaf was estimated by the quantitative analyses of energy dispersive X-ray spectroscopy (EDX) equipped with a TEM (JEM-ARM200F, JEOL *Ltd.*, Japan) and X-ray photoelectron spectroscopy (XPS) (PHI5000, ULVAC-PHI, *Inc.*, Japan). The amount of residual Ag in a NPG leaf was below 1.0 at.% (Supplementary Tables 1 and 2). The diameter of pores in the NPG sample ranges from 10 to 100 nm. For example, the pores with the diameter of 40–60 nm are confirmed in the typical TEM images in Supplementary Figure 1 and the inset image of Fig. 1a. As we mentioned in Supplementary Note 3, the nanofacet dynamics were self-activated in the reaction environment on {110} surface in a convex area, not only in a concave area. This result indicates that the pore size and morphology are not crucial factors for the catalytic chemical reaction, although the number of active sites, or the active surface area is important to total reactivity. The dependence of pore size in NPG to catalytic activity was reported by Fujita *et al.*[2]. According to the study by Fujita *et al.*, the NPG samples with the smaller pore size (6, 15 and 28 nm) have the higher activity for CO oxidation reaction than those with large pore size (40 nm). Therefore, the samples in our study can be regarded as an ordinary NPG sample: Our experimental data are representative of common NPG. The surface of as-prepared NPG leaves was slightly contaminated with carbon, as observed by TEM under vacuum (Supplementary Figure 2) and XPS (Supplementary Table 2).

Catalytic performance of NPG in CO oxidation reactions is already well described[2–8]. NPG is generally prepared by two dealloying methods in concentrated nitric acid with (as in this study) and without applied anodic potential. NPG catalyzes CO oxidation at room temperature regardless of the preparation processes[3]. Furthermore, as-prepared NPG exhibits high activity for CO oxidation at room temperature without any pretreatment[3]. Additionally, several factors such as the ligament size and composition of reactant gases affect the activity[2]. For instance, smaller ligament sizes result in higher activity (Supplementary Figure 6 of ref. 2), and NPG shows higher activity when the CO/O_2 ratio is 1/18 compared with the ratios from 4/1 to 1/16 (Supplementary Table 1 of ref. 4). In ETEM observations, the partial pressures of CO and O_2 are set lower than the corresponding partial pressures during catalytic activity measurements. The pressure gap is sometimes a critical problem in ETEM analyses for practical catalysts. However, ozone-treated NPG exhibited an affinity to oxidize CO to CO_2 when subjected to pulses of CO (maximum P_{CO} = ~4 Pa (0.03 Torr)) at 150 °C, as can be seen in Fig. 2b of ref. 4. It is also known that AuNP catalysts are active for low-temperature CO oxidation at lower partial pressures of CO and O_2[5–7]. Furthermore, we performed atomic resolution ETEM in the higher pressure of gas that was ten times higher (1000 Pa) than that in the main text (100 Pa) at the technical limit to confirm the reproducibility of ETEM observation (Supplementary Figure 3). The pressure reached about 1% of atmosphere pressure, so we think that we simulated well real catalysis in ETEM. Hence, the previous studies as well as our supplemental data (Supplementary Figure 3) strongly support that NPG, without any pretreatment, acts as a catalyst for CO oxidation under ETEM observation conditions (1 vol.% CO/air (CO/O_2 ratio = 1/20), partial pressure of CO, P_{CO} of 1 Pa).

The NPG from a commercially available Au-Ag alloy leaf may contain elements other than Au and

Ag though elemental analyses (TEM-EDX and XPS) could not detect other elements within the accuracy of the instrucments. In order to obtain a chemically well-defined NPG sample, we prepared a Au-Ag starting alloy by melting pure Au and pure Ag according to the process described by B. Zugic, *et al.*[4] (see Supplementary Figure 4). After dealloying of the starting alloy, the NPG specimens were observed by ETEM (Titan 300 kV, 4 A cm^{-2}, Ceta2 camera 2 fps). The dealloying process was identical for the NPG from a commercially available Au-Ag alloy leaf in the present study. TEM confirmed the ordinary morphology of NPG in the dealloyed sample (Supplementary Figure 5). As is demonstrated in Supplementary Figure 6, ETEM results showed that the stable nanofacet was well reproduced on {110} in O_2 (Supplementary Figure 6a), while surface atoms are moved on the {110} surface in the reaction environment of CO/air (Supplementary Figure 6b). The elemental analysis was performed on the Au-Ag starting alloy by XPS and ICP-AES (inductively coupled plasma atomic emission spectroscopy, ICPE-9820, Shimadzu. Co, Japan). The results of XPS analysis are summarized in Supplementary Table 3. The atomic ratio of Au-Ag starting alloy was identified to be 31/69 by ICP-AES analysis. No impurities were detected within the detection limits of the apparatuses. Hence the ETEM observation on the ordinary NPG sample in text is well reproduced in a chemically well-defined NPG sample. The preparation process for the well-defined sample was described as follows.

1. A pure Ag grain of 99.9999% in purity (The Nilaco Co. Tokyo) and a pure Au grain of 99.99% in purity (The Nilaco Co. Tokyo) were weighed to prepare an Ag-Au alloy of 60 wt. % Ag in nominal composition.
2. After weighing, both the pure Ag and Au grains were rinsed in acetone and ethanol by ultrasonic cleaning.
3. The pure Ag and Au grains were melted in a vacuum arc-melting furnace (ACM-01, Daia Vacuum Co.)[9]. The base pressure of the furnace was 8×10^{-6} Torr. Introducing Ar gas into the furnace, the pure Ag and Au grains were melted on a water-cooled copper crucible by a plasma arc of argon under an applied voltage of 20 V and a current of 250 A for 180 s. The resulting button-shaped ingot was then re-melted up-side down twice in the aforementioned melting process for homogenization (Supplementary Figure 4).
4. The ingot was then cut into pieces by using an electric discharge cutting machine (AQ325L, Sodick CO.). The cutting speed was 0.7 mm min^{-1}.
5. After polishing the surface of the pieces with emery papers (#1600), the pieces were rinsed in acetone and ethanol by ultrasonic cleaning.
6. A piece of the Ag-Au alloy was put in a quartz tube, the quartz tube was evacuated and sealed with Ar of 0.01 MPa. The piece of Ag-Au alloy in the sealed tube was annealed at 875 °C for 140 h for further homogenization.
7. The annealed piece was cut into small pieces of 1 to 2 mm in edge length by the electric discharge cutting.
8. After mechanically thinning a small piece up to 20 μm in thickness, the piece was dealloyed in 70% HNO_3 *aq.* for 3 h. The final piece was rinsed in distilled water.
9. The final piece was crushed in an agate mortar and supported on a carbon-coated micro-grid for (E)TEM observations. The results are shown in Supplementary Figures 3 and 6.

For the accurate estimation of turnover frequency (TOF) in a heterogeneous catalyst, the number density of catalytically active sites is essentially needed. Given the identification of the active site in this study, the previous estimations for NPG such as 0.001 to 0.5 s^{-1} (~3 s^{-1})[2,3,8] need to be corrected to estimate the number density on the entire NPG surface in future studies.

Supplementary Table 1: Quantitative analyses of atomic composition by transmission electron microscopy-energy dispersive X-ray spectroscopy (TEM-EDX).

Sample	Composition[1)]	
	Au (at.%)	Ag (at.%)
Au-Ag leaf as-obtained	26.6	73.4
NPG as-dealloyed	99.1	0.9

[1)] The atomic compositions of Au and Ag were estimated from the peak area of Au Mα and Ag Lα, respectively.

Supplementary Table 2: Quantitative analyses of atomic composition by X-ray photoelectron spectroscopy (XPS).

Sample	Au (at.%)	Ag (at.%)	C (at.%)	N (at.%)	O (at.%)
Au-Ag leaf as-obtained	16	42	31	< 1	8
Au-Ag leaf after Ar etching[1)]	29	70	< 1	< 2	1.2
NPG as-dealloyed	34	1	43	2	18
NPG after Ar etching[1)]	97	0.5	< 1	< 1	2.5

[1)] The samples were pretreated by etching with a low-energy Ar gas cluster ion beam (Ar-GCIB) (5 keV) to remove organic contamination from the surface.

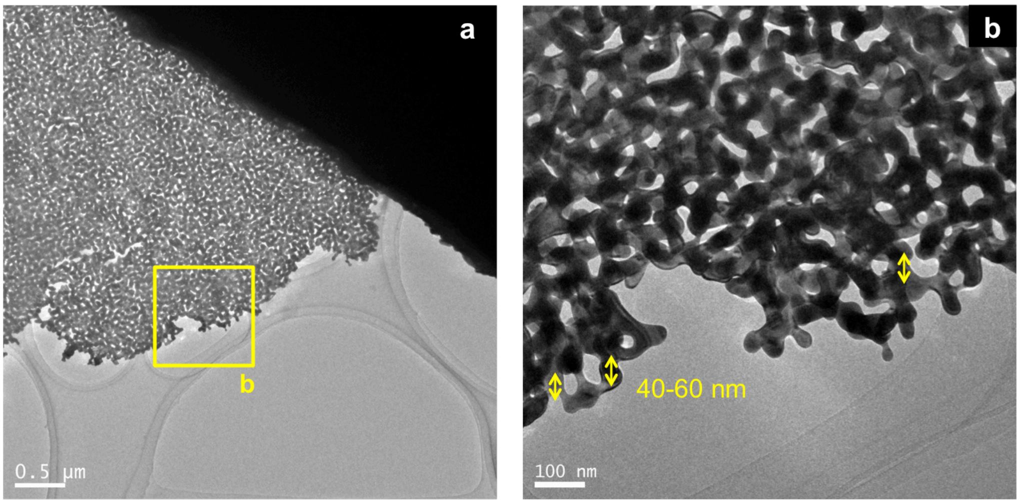

Supplementary Figure 1: Typical TEM images of the as-dealloyed NPG.

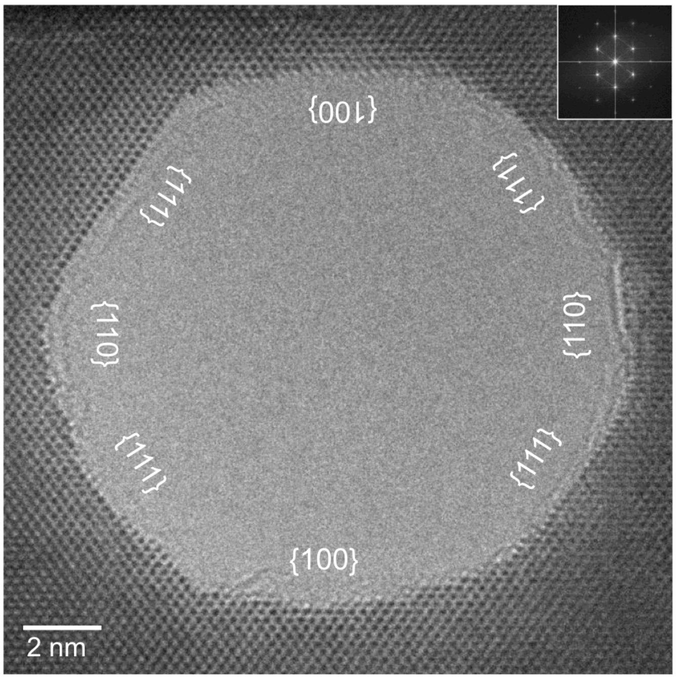

Supplementary Figure 2: A typical pore of nanoporous gold (NPG) observed by transmission electron microscopy (TEM) under vacuum (1.9×10^{-5} Pa). Fourier transform pattern of the TEM image (inset) shows the single crystallinity around the pore.

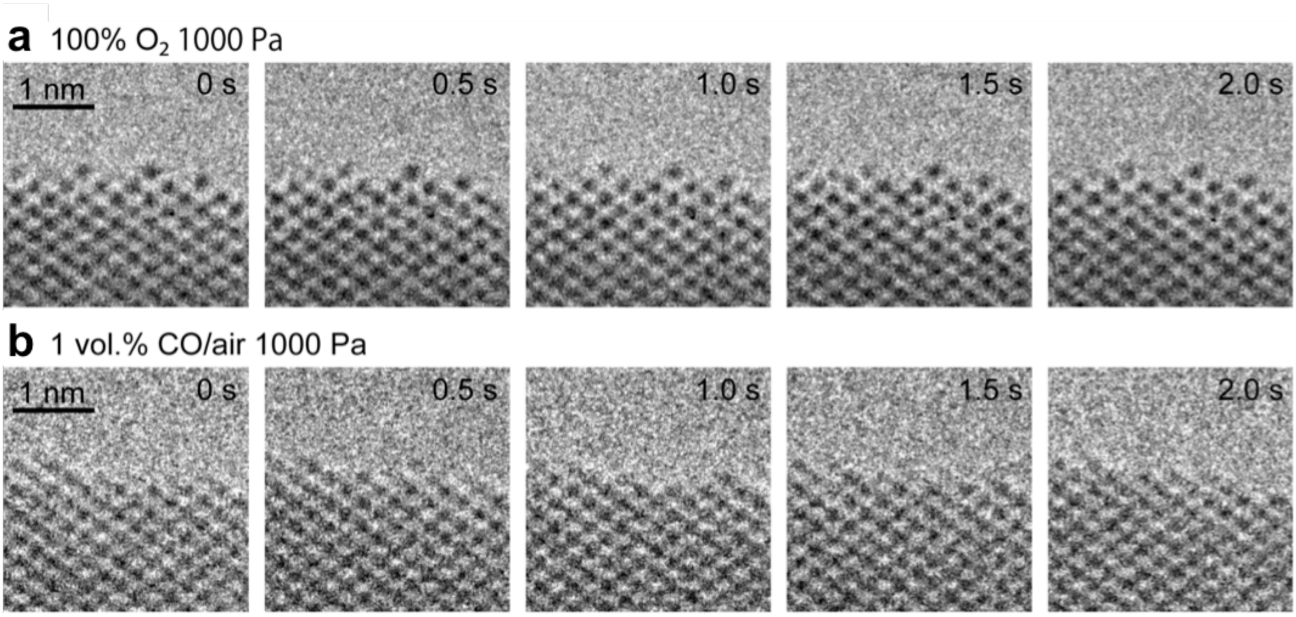

Supplementary Figure 3: ETEM images of the {110} surfaces of NPG in the high pressure of 1000 Pa. **a** O_2 and **b** 1 vol.% CO/air. The nanofacet was observed at a higher pressure of O_2 (1000 Pa) as that observed in at lower pressure of O_2 (100 Pa) (Fig. 1b and Supplementary Figure 8b). Therefore, the formation of the nanofacet on {110} is reproduced in the range of O_2 gas pressure from 100 to 1000 Pa. At 1 vol.% CO/air at the higher pressure (1000 Pa), the surface atoms were displaced continuously and the stable nanofacet was not confirmed (Supplementary Figure 3b) as at the lower pressure of 1 vol.% CO/air (100Pa) (Fig. 1a and Supplementary Figure 8a). The surface atoms appear to move more at higher pressure (1000 Pa) than at lower pressure (100 Pa). For taking atomic resolution images even at the high pressure gas, 300 keV electrons (4 A cm^{-2} s^{-1} in electron current density) were employed. 300 keV electrons enhanced electron irradiation effects that are described in both O_2 and 1 vol.% CO/air in text. The instability of surface atoms in CO/air is likely explained by the breaking of Ag-O bonds by CO gas and electron beam irradiation. The surface atoms appear to be displaced more vigorously and the nanofacet structure could not be recorded in 1 vol.% CO/air (1000 Pa).

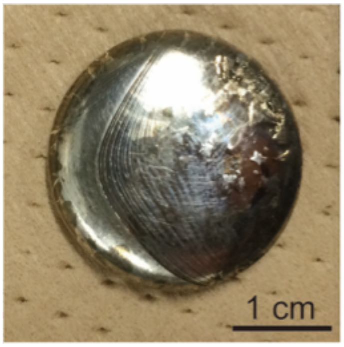

Supplementary Figure 4: The ingot of Ag-Au alloy formed by arc-melting of pure Ag and pure Au.

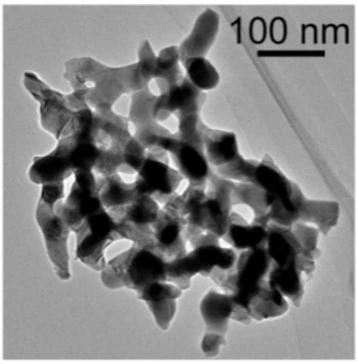

Supplementary Figure 5: TEM image of the NPG specimen prepared from pure Ag and pure Au by arc-melting followed by the dealloying process.

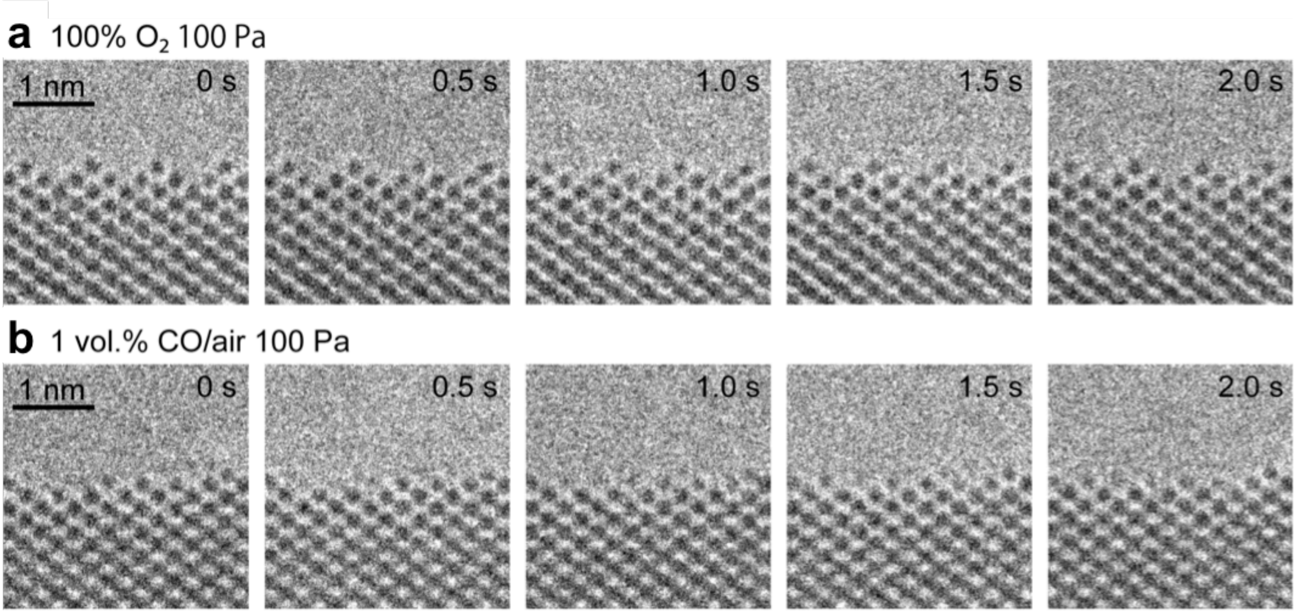

Supplementary Figure 6: ETEM observation of a chemically well-defined NPG sample in O_2 (100Pa) and 1 vol.% CO/air (100Pa). ETEM observation on the ordinary NPG sample in text is well reproduced: The formation of the stable nanofacet on {110} in O_2 in **a** and displacement of surface atoms on {110} in 1 vol.% CO/air in **b**.

Supplementary Table 3: Quantitative analyses of atomic composition by X-ray photoelectron spectroscopy (XPS) of the chemically well-defined NPG sample from a Au-Ag starting alloy formed by melting pure Au and pure Ag.

Sample	Au (at.%)	Ag (at.%)	C (at.%)	N (at.%)	O (at.%)
Well-defined Au-Ag leaf after Ar etching[1)]	31	69	< 1	< 3	< 0.5
Well-defined NPG after Ar etching[1)]	94	1.9	< 2	< 3	4.1

[1)] The samples were pretreated by etching with a low-energy Ar gas cluster ion beam (Ar-GCIB) (5 keV) to remove organic contamination from the surface.

Supplementary Note 2. ETEM observation, image simulation and image analysis.

A part of an as-prepared NPG leaf was placed onto a Cu grid with a holey carbon supporting film for ETEM observation. A Au foil (The Nilaco Corporation, purity 99.9+%) was also used as a reference sample. A double mesh Cu grid in the absence of a carbon supporting film was used for ETEM observations of the Au foil. NPG was observed using an ETEM apparatus (Titan ETEM G2, FEI company, USA) with a spherical aberration corrector (Cs-corrector) of the objective lens, a monochromator and a K2 IS Direct Detection Camera (Gatan *Inc.*, USA). To minimize electron beam damage to NPG, the accelerating voltage and electron current flux during ETEM observations were set at 80 kV and 4 A cm^{-2}, respectively, otherwise noted. The spherical aberration of the objective lens was corrected to below 1 μm using the Cs-corrector. ETEM images were acquired at a frame rate of 40 fps in counting (summit) mode using a K2 camera. Three gases, 1 vol.% CO/air (1 vol.% CO, 21 vol.% O_2, 78 vol.% N_2) (100 Pa), 100% O_2 (100 Pa) and 100% CO (100 Pa) were introduced into the specially designed environmental cell of the ETEM apparatus at room temperature. Pre-treatment of the sample such as ion cleaning was not performed to prevent any surface structural changes to the NPG or the oxidation of residual Ag species.

TEM images of model structures, constructed in Supplementary Note 4 were simulated using Mac-TempasX software (Total Resolution, USA). For image simulation, the parameters of accelerating voltage, spherical aberration coefficient of the objective lens, chromatic aberration coefficient of the objective lens and the radius of the objective aperture were set to 80 kV, 500 nm, 1.4 mm and 10 nm^{-1}, respectively. In the simulation, the sufficiently large size of the supercell was used to avoid termination errors. The effect of electron scattering on gas molecules was not considered, because it just degrades the clearness of ETEM images[10,11].

The time-dependent distance between two atomic columns on the surface (Fig. 2a, b) was estimated from the following procedure. In an image at time, t in a series of *in situ* images after 4×4×4 binning (frame rate: 10 fps), we assumed that the intensity profile of an individual atomic column can be fitted with a Gaussian function as follows.

$$I(x) = -\sum_{\mathrm{i}=1}^{\mathrm{N}} \frac{I_{\mathrm{i}}}{\sqrt{2\pi \cdot \Delta x_{\mathrm{i}}^2}} \exp\{(-(x - x_{\mathrm{i}})^2)/(2\Delta x_{\mathrm{i}}^2)\} + BG$$

Here, x_i, I_i and Δx_i represent the position, the peak intensity parameter and the intensity deviation of an i-th atomic column, respectively. BG represents the averaged background intensity in the outside of a specimen, or in a gas. The number of atomic columns, N was taken as N = 2 in the analysis. After least square fitting, the distance between the i-th and j-th atomic columns at time t, $D(t)$ is readily obtained as $|x_j - x_i|$, as is summarized in Fig. 2c, d. Distribution of $D(t)$ for time intervals of 100 ms in Fig. 2e are useful to indicate the mutual stability of the i-th and j-th atomic columns as a function of time. When $D(t)$ holds a certain value, say D_0 for an extended period of time, the integration time in Fig. 2e at D_0 increases. Therefore, the distribution of $D(t)$ in Fig. 2e implies the fractional time of a specific surface structure in one cycle of the catalyzed chemical reaction.

Supplementary Note 3. Morphology changes under various environments in a convex area of NPG.

The surface structures on {111} and {110} facets in a concave area (Fig. 1) are also reproduced in a convex area of NPG, as is shown in Supplementary Figure 7. At higher magnification, the structural characteristics on {110} in 1 vol.% CO/air (100Pa), 100% O_2 (100 Pa) and 100% CO (100 Pa) can be observed (Supplementary Figure 8). The nanofacet dynamics were self-activated in the reaction environment in a convex area, as well as a concave surface (Fig. 2a, b).

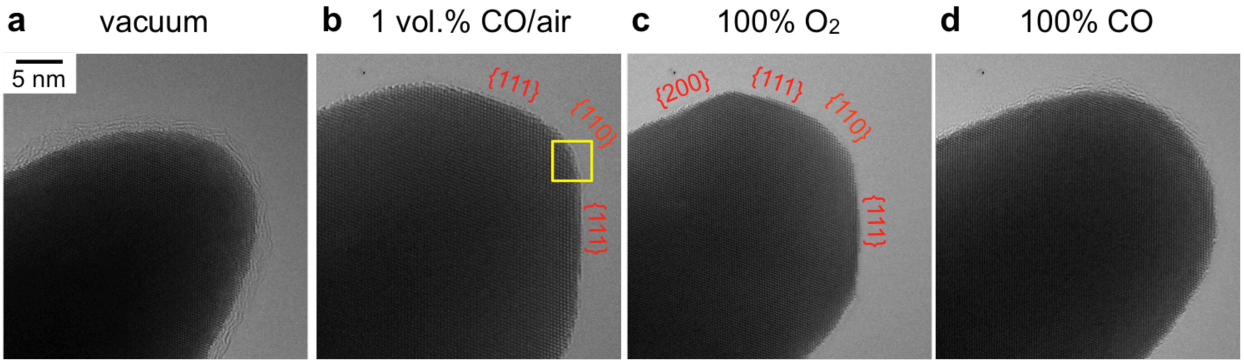

Supplementary Figure 7: Morphology of a convex area in NPG under different environments. **a** vacuum, **b** 1 vol.% CO/air (100 Pa), **c** 100% O_2 (100 Pa), and **d** 100% CO (100 Pa).

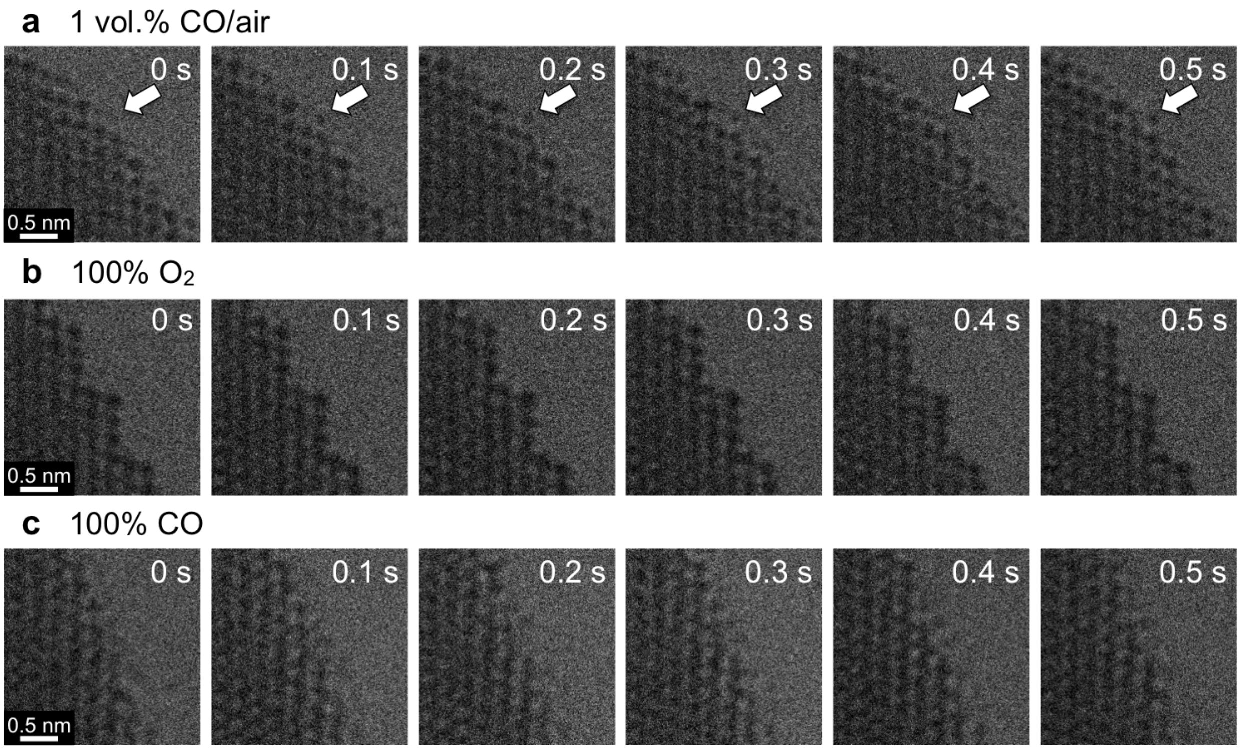

Supplementary Figure 8: Higher magnification observations of a nanofacet in a convex area, indicated by the yellow square in Supplementary Figure 2 under various environments. Arrows in **a** indicate the dynamic nanofacet in the reaction environment.

Supplementary Note 4. Atomic dynamics on the top atomic column observed by ETEM

A series of two-dimensional projection of a specimen in a gas environment can only be observed with time by *in-situ* atomic resolution ETEM. It is also technically impossible to analyze the chemical composition of individual atomic columns *in-situ* and in a gas environment. Nevertheless, by changing the gas environment, we elucidate as described in text that the peculiar atomic columns are self-organized along the direction of the projection and that the atoms on the specific atomic columns move in the reaction condition. Supplementary Figure 9, as a reproduction of Fig. 2a in the text reveals that the atoms in the columns successively moved, finally causing the movement of the atomic columns. Supplementary Figure 9b shows the image intensity of the two atomic columns along a white dotted line. While the image intensity of the second top column remains the similar with time, that of the topmost column fluctuates with time. This indicates that an entire atomic column that consists of a large number of atoms does not moved instantaneously at the reaction condition.

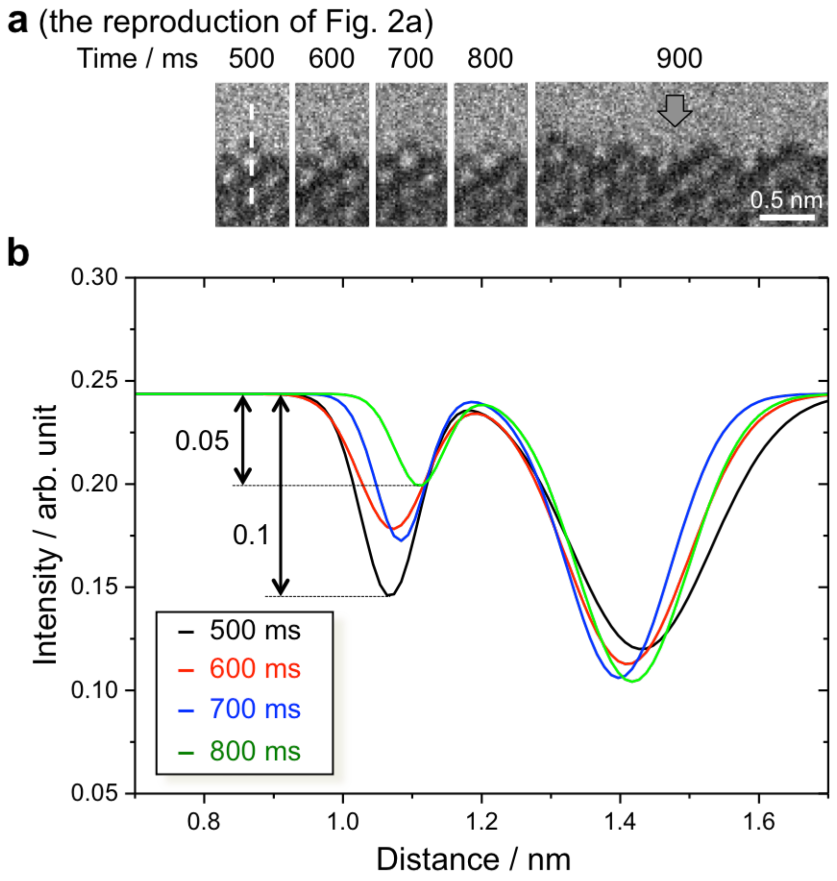

Supplementary Figure 9: Change of image intensity at the atomic columns with time. The intensity profile is shown along the white dotted line in **a**, the reproduction of Fig. 2a in text. In **b**, the image intensity of the top column, a shallower minimum in the left-hand side is varied with time (500, 600, 700 and 800 ms), while that in the second topmost atomic column, a deeper minimum in the right-hand side remain similar. Note that the distance between the intensity maximum of the topmost and the second topmost atomic columns, or the distance between the columns is unchanged within the experimental error of 0.097 nm. This indicates that Ag atoms are moved successively from and to the top atomic column with time.

Supplementary Note 5. Models for nanofacet with Ag and O.

Several Au/AgO_x surface models were constructed for a nanofacet on {110} and extended AgO atomic layers on {111}. Structural models for the nanofacet were derived from the hypothetical nanofacet of pure gold as a basis, involving silver and oxygen atoms. For the structure optimization and energy calculation for energetically favorable structures in Au/AgO_x, Vienna *Ab-Initio* Simulation Package (VASP)[12] was used for the density functional theory (DFT) calculations. The exchange-correlation energy and potential are described by the generalized gradient approximation in the form of Perdew-Burke-Ernzerhof (PBE)[13]. Plane waves with a cutoff of 400 eV were used for projector augmented wave (PAW) potentials[14,15]. The criteria for the convergence are residual forces less than 0.02 eV $Å^{-1}$. The {110} surfaces with AgO_x or AuO_x deposition are modeled in DFT calculations by a thirteen-layer slab with a vacuum region of 1.5 nm thick. The top nine layers were relaxed while the four bottom layers were fixed at the bulk positions in fcc gold. A 3×1 Au {110} supercell was used with Monkhorst-Pack (MP) 3×5×1 *k*-point mesh. The {111} surfaces with single and double layer AgO_x cluster deposition are modeled by four-layer slabs with a vacuum region of 1.2nm thick and three-layer slabs with a vacuum region of 1.5 nm thick, respectively. The bottom two layers are fixed at the bulk positions in fcc gold. A 7×7 Au {111} supercell was used with MP 1×1×1 *k*-point mesh. Spin polarization is considered during all the calculations.

Since *in-situ* ETEM observation, especially with a Cs-corrector of the objective lens can experimentally provide us with not chemical composition of individual atomic columns but the position of atomic columns accurately, the estimation of the distance between atomic columns by both ETEM observation and *ab-initio* calculation is useful for identifying the atomic species on the surface. In the framework of fcc structure, the estimation of the distance between atomic columns can lead to the distance between the associated atoms not in the projection plane (*i.e.*, the ETEM image plane) but in 3D. The distances between the top atomic column and that immediately below, *D* was calculated for the models. Supplementary Figure 10 summarizes the 3D view of the models and the corresponding *D*. The distance remained ~0.285 nm by substituting gold atomic columns with silver atomic columns. However, when oxygen atoms are adsorbed at the energetically favorable sites near the Ag and/or Au atomic columns, *D* is significantly increased up to about 0.32–0.35 nm. We confirmed that the peculiar nanofacet is stabilized in a pure oxygen environment. The inter atomic-column distance between the topmost and second topmost atomic columns cannot be accounted for by neither pure Au nor pure Ag. *Ab-initio* energy calculation indicates the model with Ag atoms on the topmost atomic column to be energetically favorable. The measured interatomic distances agree with those calculated by energy calculation. In addition, there are possible 3D nanofacet models with much more complex Ag-O configurations. Though the complex models cannot be ruled out, the typical nanofacet model with Ag on the top column is discussed in text.

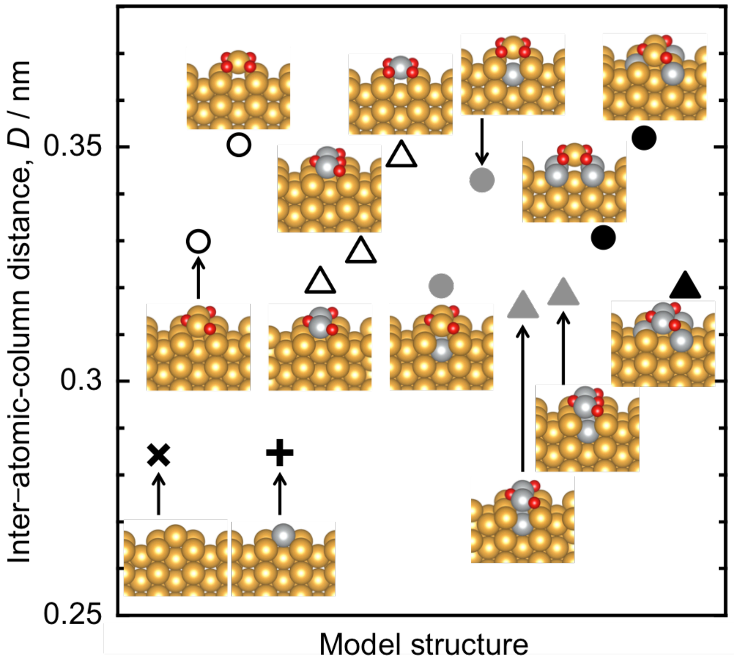

Supplementary Figure 10: Model structures for nanofacets with the corresponding inter-atomic-column distance, *D*. (1) Basic models without oxygen. ×: Pure gold, +: Ag at the top column. (2) Surface Ag oxide models. △: Ag at the top column with oxygen, [gray ▲]: Ag at both the top column and immediately below the top column with oxygen, ▲: extended Ag substitution with oxygen. (3) Sub-surface Ag models. [gray ●]: Ag at the column immediately below the top column with oxygen, ●: extended sub-surface Ag with oxygen. (4) Surface Au oxide models. ○: Pure gold with oxygen at different adsorption sites.

Supplementary Note 6. Pure gold foils in the reaction environment for NPG catalyst.

As reference, the surface of a pure Au foil was observed in 1 vol.% CO/air (100 Pa) to clarify the distinction between a NPG leaf with residual Ag and a pure Au foil (without elemental Ag). As is shown in Supplementary Figure 11, {110} facets were not extended in the environment and consequently no nanofacet was self-activated. Though multisteps were observed on {111} (Supplementary Figure 11b), the displacement of the surface atomic columns was hardly observed.

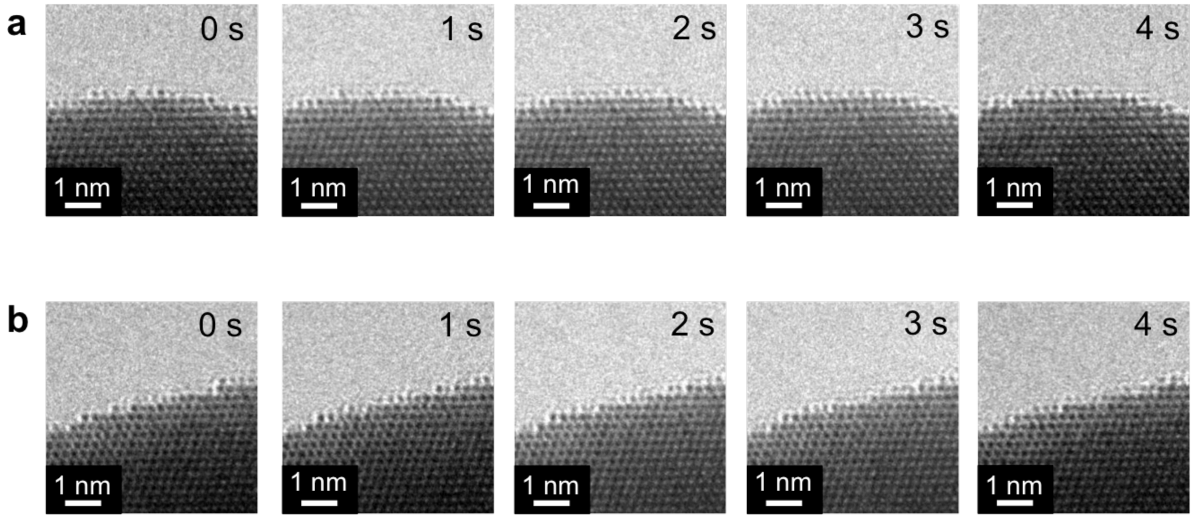

Supplementary Figure 11: Surface structure of a pure Au foil under the same reaction environment as for the NPG catalyst. **a** A part of the surface near {111} and **b** a different surface part having multiple steps.

Supplementary Note 7. Identification of the layered surface structures as Ag oxide.

Extended atomic layer structures[4] were self-activated on the surface of NPGs in both pure O_2 and reaction environments. However, the extended structures were rarely observed in NPG in the environments in this study for the oxidation of CO at room temperature. Apparently, the extended structure was unstable under vacuum (Supplementary Figure 12). As shown in Supplementary Figure 12, a double atomic layer structure, which spontaneously formed in 1 vol.% CO/air (100 Pa), was gradually destroyed under vacuum when subjected to electron beam irradiation. Interestingly, the double atomic layer structure was regenerated in 100% O_2. The change in stability in different environments suggested that the extended structure is silver oxide in nature, as in a previous suggestion[4]. An extended structure of size smaller than a few nanometers, say a nanoparticle, was structurally unstable even in a reaction environment, especially at the perimeter interface between the nanoparticle and the NPG matrix (Supplementary Figure 13). It is noteworthy that the atoms at the perimeter interface were vigorously displaced in the reaction environment, as in the nanofacets. In the double atomic layer structure, the spacing between the first and second atomic layers was measured as 0.32 nm (Supplementary Figure 15). This spacing cannot be explained by the lattice spacing of metallic Au or Ag, however, this is consistent with Ag oxide. *Ab-initio* calculations confirmed that the model structure is suitable for the double atomic layer structure (Supplementary Figure 15b). The single layer structure is also likely to be Ag oxide (Supplementary Figure 14).

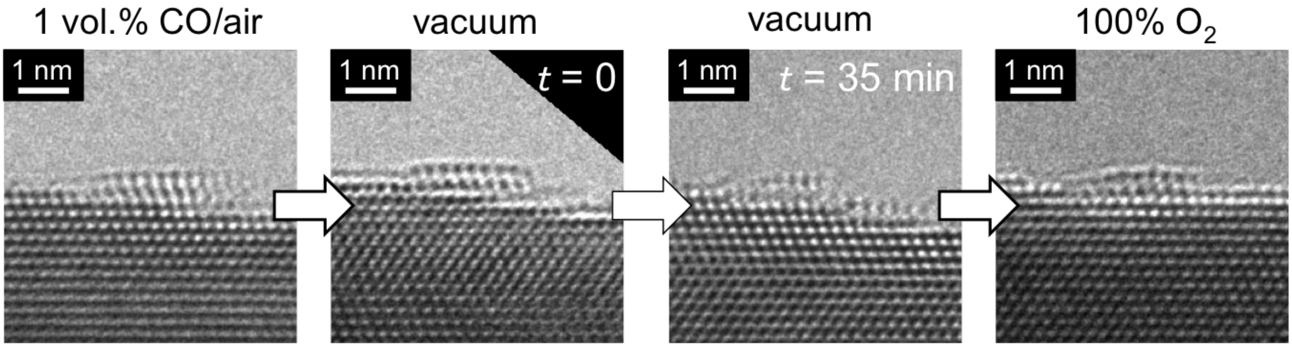

Supplementary Figure 12: Stability of a double atomic layer structure on the surface of NPG. The extended structure, observed in 1 vol.% CO/air (100 Pa) became unstable under vacuum when subjected to electron beam irradiation. The extended structure was regenerated in O_2 (100 Pa).

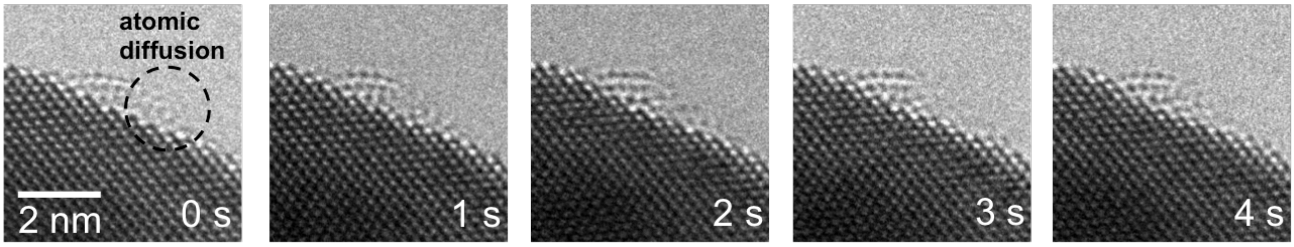

Supplementary Figure 13: Atomic dynamics at the perimeter interface between a double atomic layer structure on the surface of NPG in 1 vol.% CO/air (100 Pa).

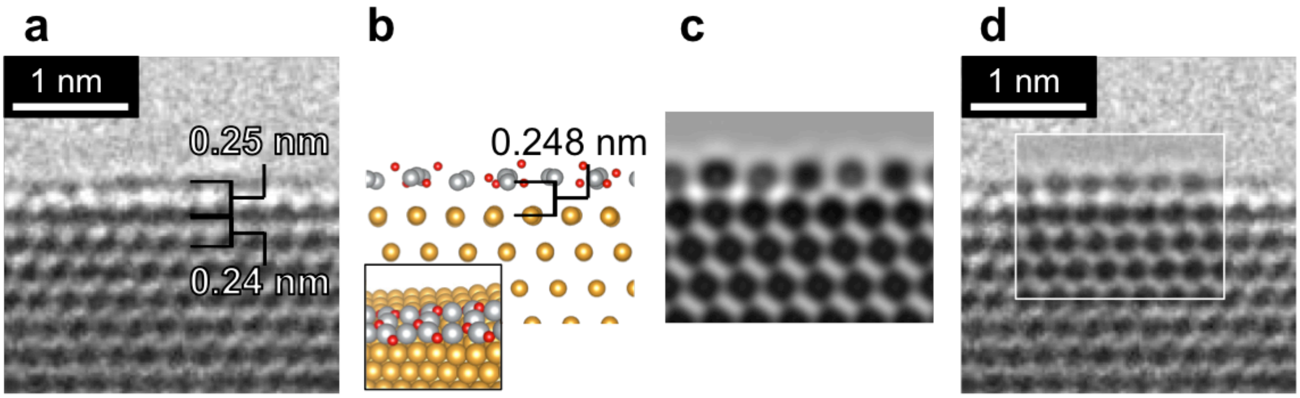

Supplementary Figure 14: A single atomic layer structure in 1 vol.% CO/air (100 Pa). **a** ETEM image, **b** model structure after *ab-initio* calculations and **c** simulated image. In **d**, the simulated image was overlaid onto a part of the ETEM image.

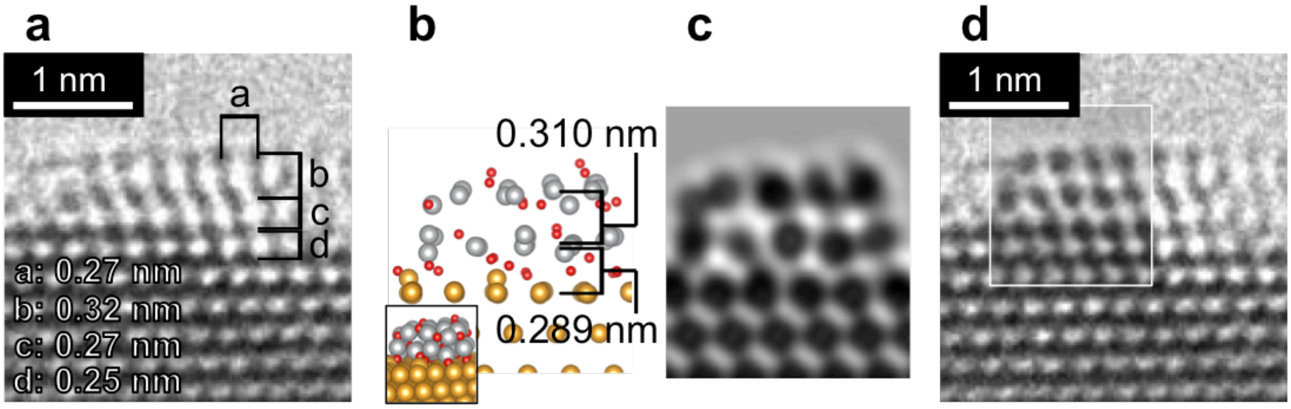

Supplementary Figure 15: A double atomic layer structure in 1 vol.% CO/air (100 Pa). **a** ETEM image, **b** model structure after *ab-initio* calculations and **c** simulated image. **d** Simulated image was overlaid onto a part of the ETEM image.

Supplementary Note 8. Surface dynamics in ETEM observations of low electron current density.

As is shown in Figs. 1 and 2, nanofacets on {110} are self-activated in pure O_2 and a reaction environment. Surface dynamics were induced when subjected to the reaction environment. The nanofacet top sites are frequently relocated in the presence of the reaction environment, as is demonstrated in Fig. 2a. Supplementary Figure 16 revealed that displacement occurs even at a low electron current density (0.4 A cm^{-2}). This result supports that nanofacet dynamics are an intrinsic phenomenon without the aid of electron beam irradiation.

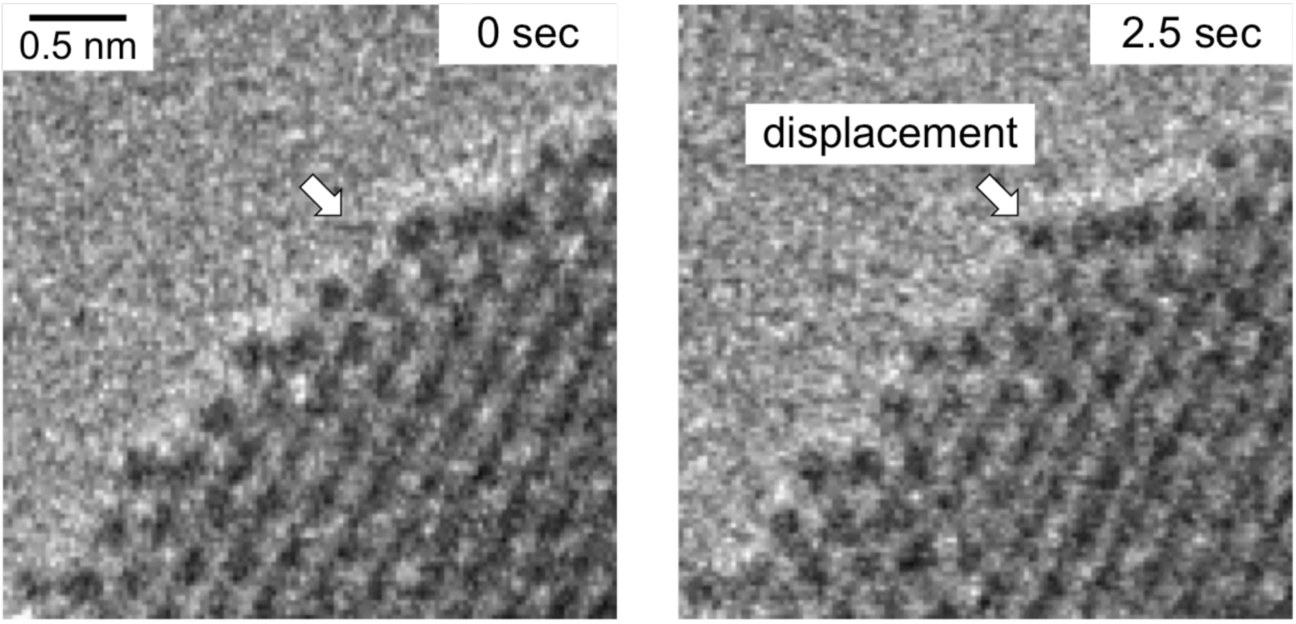

Supplementary Figure 16: NPG surface atom displacement in the reaction environment (1 vol.% CO/air (100 Pa)) observed when subjected to a low electron current density of 0.4 A cm^{-2}.

Supplementary Note 9. Note on electron irradiation-induced artifacts in 100% O_2 and 100% CO.

Surface structures in gases are potentially modulated by high-energy electron beam irradiation. Artifacts by electron irradiation in ETEM observations are summarized for future study of NPGs. In the environments, including O_2, amorphous layers were often observed on the surface of NPG, as is seen in TEM and High-Angle Annular Dark-Field-Scanning Transmission Electron Microscopy (HAADF-STEM) images (Supplementary Figure 17). As is shown below, it is confirmed that the amorphous layers were formed only in the heavily electron-irradiated areas and are not intrinsically responsible to catalytic performance.

First, the thin amorphous layers were formed in 100% O_2 on the surface of NPG by electron irradiation (80 keV, 4 A cm^{-2} for 19.5 h). The amorphous layers were then analyzed by STEM-electron energy loss spectroscopy (EELS) at 300 keV (Supplementary Figure 18) and STEM-EDX at 200 keV (ARM200F, JEOL) (Supplementary Figure 19) under vacuum. In Supplementary Figures 18 and 19, the amorphous layers are indicated by white arrows in the STEM images that are acquired at a low contrast mode. In EELS (Supplementary Figure 18), O-K edges were clearly detected at about 530 eV in amorphous layers on NPG, while neither the Ag-$M_{4,5}$ edge (~400 eV) or C-K edge (~280 eV) were detected. The low-loss spectra from the amorphous layers cannot be identified because of the broadness. The EDX spectrum (Supplementary Figure 19), having sufficient signal, was not detected from the thin amorphous layers. Metal species, either Ag or Au, were not measured within the detection limit of the EDX apparatus. Nevertheless, given the EELS data above, the amorphous layer can be identified not to be carbon but silver oxides. Additionally, the surface of Au films can be oxidized to Au_2O_3 in the presence of activated oxygen gas such as plasma[16] and ozone[17]. NPG includes an abundance of under-coordinated Au atoms[18] and the unstable Au atoms may be more easily oxidized in the presence of activated oxygen gas than well-coordinated Au atoms in pure Au films. It is therefore concluded that the amorphous layers of Ag oxide as well as Au oxide are formed by oxygen that is activated by electron beam irradiation. The amorphous layer is not the essential surface nanostructure for catalysis of NPG for CO oxidation.

In 100% CO, previous works reported that the surface of NPG was covered with carbon because of the dissociation of CO[2,19]. In our experiments, however, carbon deposition on the surface was absent when controlling electron irradiation-induced damage (Fig. 1c). The discrepancy with previous ETEM reports[2,19] becomes evident, since the NPGs observed in this study were subjected to ETEM imaging conditions at a lower electron energy (80 keV) and lower electron current density (0.4–4 A cm^{-2}).

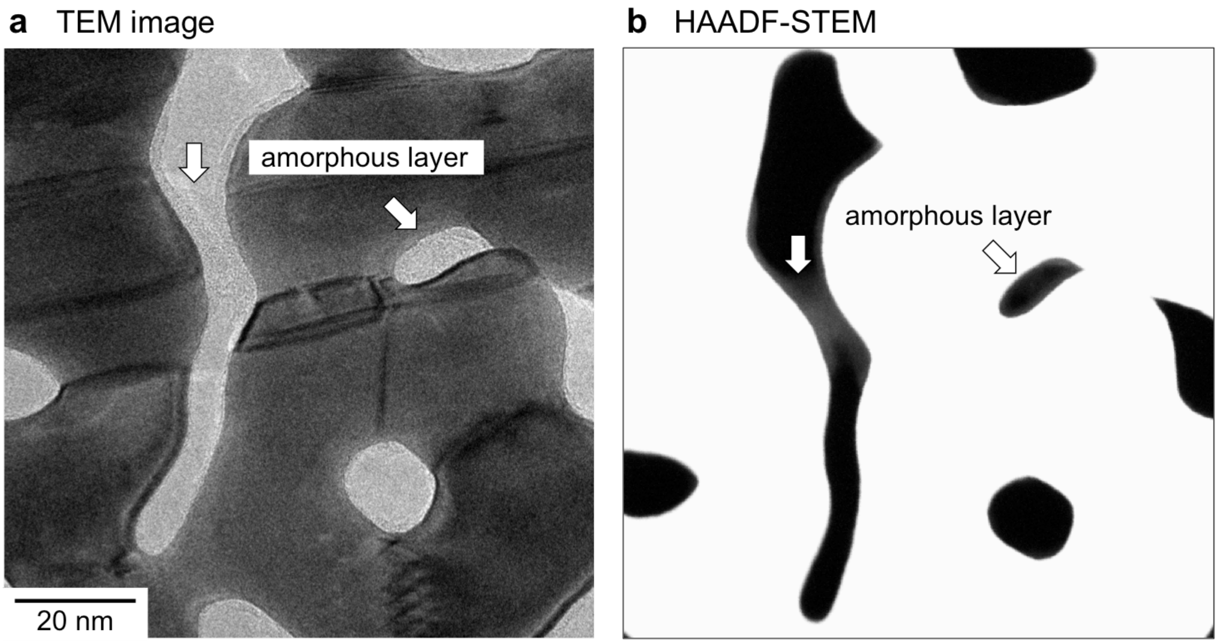

Supplementary Figure 17: TEM and high-angle annular dark-field-scanning transmission electron microscopy (HAADF-STEM) images of amorphous layers observed under vacuum after the ETEM observation in 100% O_2 (100 Pa) at 80 keV, 4 A cm^{-2} for 19.5 h.

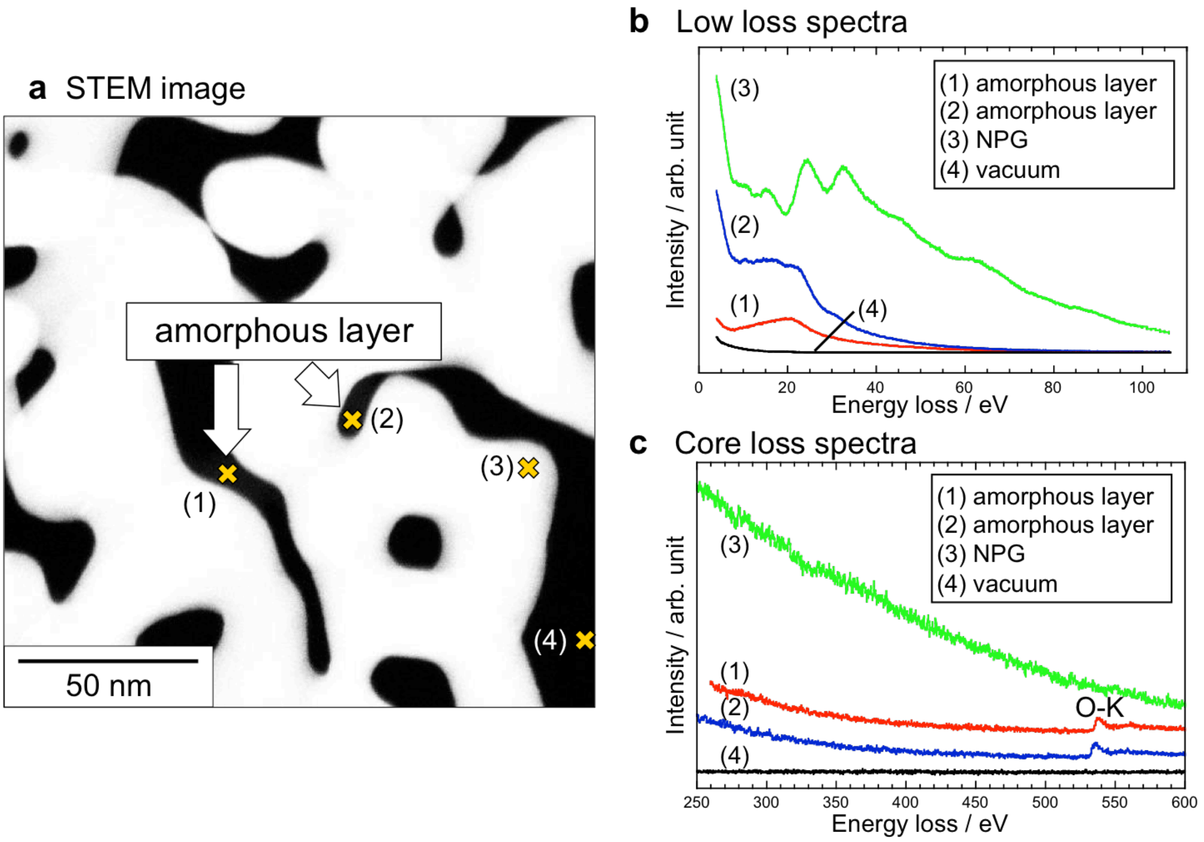

Supplementary Figure 18: Identification of amorphous layers by STEM-electron energy loss spectroscopy (EELS). **a** A HAADF-STEM image, **b** low-loss and **c** core-loss EELS spectra that were acquired at the four points in the HAADF-STEM image **a**.

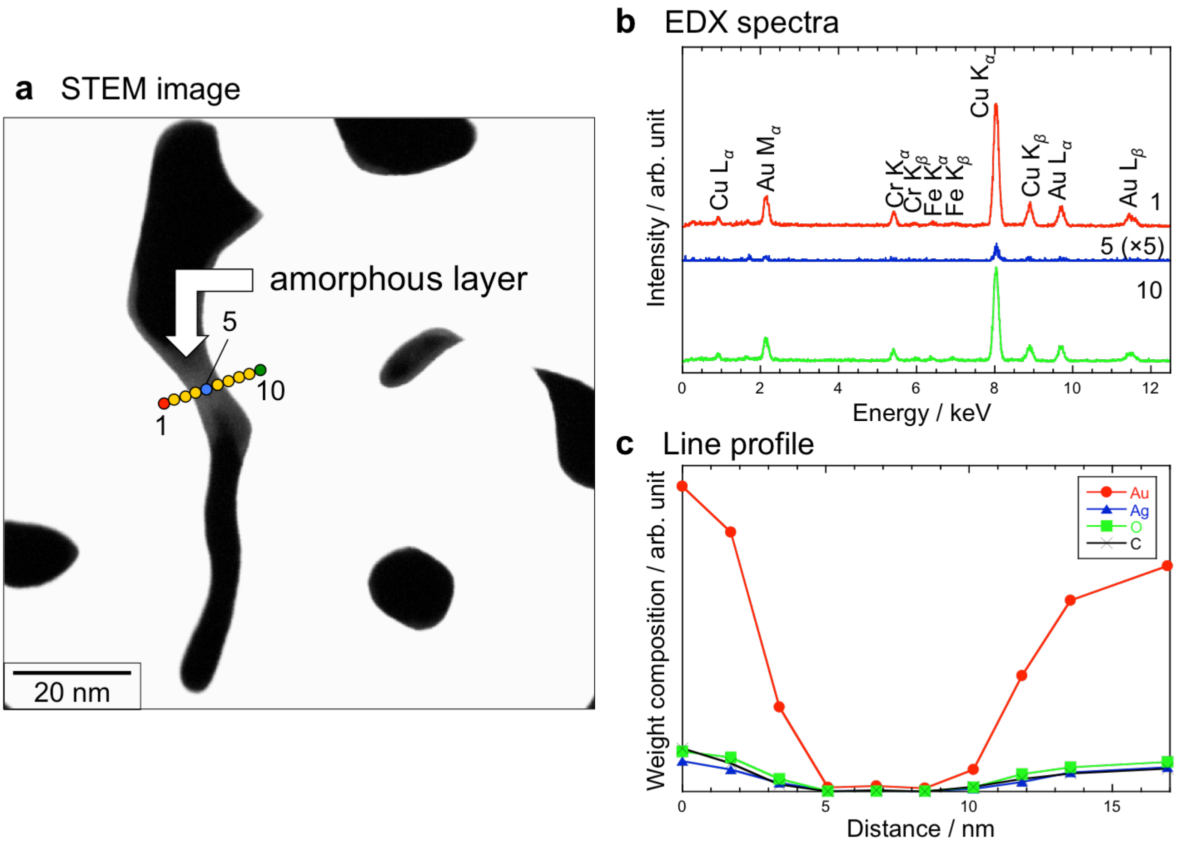

Supplementary Figure 19: Identification of amorphous layers by STEM-energy dispersive X-ray spectroscopy (EDX). **a** A HAADF-STEM image, **b** EDX spectra and **c** line profiles of Au, Ag, O and C concentrations across the line indicated in **a**. The Cu peak is attributed to the supporting Cu grid.

Supplementary Note 10. Durability of NPGs in a reaction environment.

Supplementary Figure 20 demonstrates the change in morphology of a pore in the presence of a reaction environment. Through surface diffusion in the environment, the nanofacet disappeared after a prolonged reaction time of 14 h.

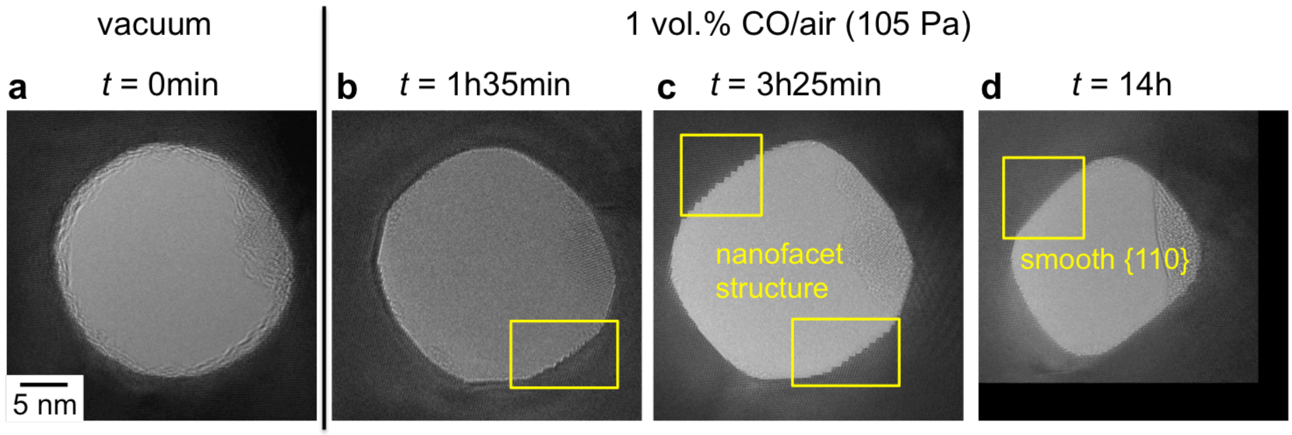

Supplementary Figure 20: Durability of nanofacets in the presence of a reaction environment (1 vol.% CO/air, 105 Pa). The nanofacets were self-activated in the reaction environment in **b** and gradually changed to the ordinary flat {110} facets after 14 h in **d**.

Supplementary References

1. Ding, Y. & Erlebacher, J. Nanoporous Metals with Controlled Multimodal Pore Size Distribution. *J. Am. Chem. Soc.* **125**, 7772–7773 (2003).

2. Fujita, T. et al. Atomic Observation of Catalysis-Induced Nanopore Coarsening of Nanoporous Gold. *Nano Lett.* **14**, 1172–1177 (2014).

3. Xu, C. et al. Low Temperature CO Oxidation over Unsupported Nanoporous Gold. *J. Am. Chem. Soc.* **129**, 42–43 (2007).

4. Zugic, B. et al. Dynamic Restructuring Drives Catalytic Activity on Nanoporous Gold-Silver Alloy Catalysts. *Nat. Mater.* **16**, 558–564 (2017).

5. Yoshida, H. et al. Visualizing Gas Molecules Interacting with Supported Nanoparticulate Catalysts at Reaction Conditions. *Science* **335**, 317–319 (2012).

6. Kuwauchi, Y., Yoshida, H., Akita, T., Haruta, M. & Takeda, S. Intrinsic Catalytic Structure of Gold Nanoparticles Supported on TiO_2. *Angew. Chem. Int. Ed.* **51**, 7729–7733 (2012).

7. Kuwauchi, Y., Takeda, S., Yoshida, H., Sun, K., Haruta, M. & Kohno, H. Stepwise Displacement of Catalytically Active Gold Nanoparticles on Cerium Oxide. *Nano Lett.* **13**, 3073–3077 (2013).

8. Wittstock, A. et al. Nanoporous Au: An Unsupported Pure Gold Catalyst?. *J. Phys. Chem. C* **113**, 5593–5600 (2009).

9. Tane, M., Hagihara, K., Ueda, M., Nakano, T. & Okuda, Y. Elastic-modulus enhancement during room-temperature aging and its suppression in metastable Ti-Nb-Based alloys with low body-centered cubic phase stability. *Acta Mater.* **102**, 373–384 (2016).

10. Yoshida, H. & Takeda, S. Image formation in a transmission electron microscope equipped with an environmental cell: single-walled carbon nanotubes in source gases. *Phys. Rev. B* **72**, 195428-1–195428-7 (2005).

11. Takeda, S., Kuwauchi, Y. & Yohida, H., Environmental transmission electron microscopy for catalyst materials using a spherical aberration corrector. *Ultramicroscopy*, **151**, 178–190 (2015).

12. Kresse, G. & Hafner, J., Ab-Initio Molecular-Dynamics for Open-Shell Transition-Metals. *Phys. Rev. B* **48**, 13115–13118 (1993).

13. Perdew, J. P., Burke, K. & Ernzerhof, M. Generalized gradient approximation made simple. *Phys. Rev. Lett.* **77**, 3865–3868 (1996).

14. Blochl, P. E. Projector Augmented-Wave Method. *Phys. Rev. B* **50**, 17953–17979 (1994).

15. Kresse, G. & Joubert, D. From ultrasoft pseudopotentials to the projector augmented-wave method. *Phys. Rev. B* **59**, 1758–1775 (1999).

16. Tsai, H., Hu, E., Perng, K., Chen, M., Wu, J.C. & Chang, Y.S. Instability of gold oxide Au_2O_3. *Surf. Sci.* **537**, L447–L450 (2003).

17. Min, B.K., Alemozafar, A.R., Pinnaduwage, D., Deng, X. & Friend, C.M. Efficient CO Oxidation at Low Temperature on Au(111). *J. Phys. Chem. B* **110**, 19833–19838 (2006).

18. Liu, P. et al. Visualizing Under-Coordinated Surface Atoms on 3D Nanoporous Gold Catalysts. *Adv. Mater.* **28**, 1753–1759 (2016).

19. Fujita, T. et al. Atomic origins of the high catalytic activity of nanoporous gold. *Nature Mater.* **11**, 775–780 (2012).
