# Supplementary material for: Self-activated surface dynamics in gold catalysts under reaction environments
Source: Nat Commun. 2018 May 25;9:2060. doi: 10.1038/s41467-018-04412-4 (PMC5970267; doi:10.1038/s41467-018-04412-4)
Supplement: Supplementary file 2 — Description of Additional Supplementary Files [file 41467_2018_4412_MOESM2_ESM.pdf]

Description of Additional Supplementary Files

File Name: Supplementary Movie 1
Description: An ETEM movie of a nanofacet in NPG in 1 vol.% CO/air (100 Pa). ETEM images in Fig. 2a were taken from this movie, and *D* in Fig. 2c was also estimated in this movie.

File Name: Supplementary Movie 2
Description: An ETEM movie of a nanofacet in NPG in 100% O_2 (100 Pa). ETEM images in Fig. 2b were taken from this movie, and *D* in Fig. 2d was also estimated in this movie.
